# Nursing and the essential public health functions framework in the Americas: reflections and perspectives

**DOI:** 10.1590/0034-7167-2025-0121

**Published:** 2026-01-26

**Authors:** Eduardo Neves da Cruz de Souza, Maria de Lourdes de Almeida, Juana Elvira Suárez Conejero, Aida Maris Peres

**Affiliations:** I Universidade Federal do Paraná. Curitiba, Paraná, Brazil; II Universidade Estadual do Oeste do Paraná. Foz do Iguaçu, Paraná, Brazil; III Universidad Nacional Autónoma de Mexico. Ciudad de México, Mexico

**Keywords:** Nurse, Professional Practice, Professional Competence, Essential Public Health Functions, Americas., Enfermería, Práctica Profesional, Competencia Profesional, Funciones Esenciales de la Salud Pública, Américas.

## Abstract

**Objectives:**

to reflect on nursing’s role in essential public health functions in the Americas, highlighting contributions, challenges, and perspectives for strengthening public health actions.

**Methods:**

this theoretical-reflective study was conducted between March and April 2025, based on Pan American Health Organization official documents, especially the 2002 and 2021 versions of the essential functions. The materials were identified by searching the Pan American Health Organization’s institutional repository, including normative and descriptive documents. The analysis was structured in four stages: historical evolution; conceptual update of the functions; impact on the Brazilian reality; and nursing contributions.

**Results:**

the updated essential functions incorporated social determinants. Nursing stands out for its strategic role in surveillance, health promotion, and public policy formulation.

**Final Considerations:**

the updated essential functions expand opportunities for nursing to reduce inequalities and strengthen resilient and inclusive health systems.

## RESUMEN

**Objetivos:**

reflexionar sobre el rol de la enfermería en las funciones esenciales de salud pública en las Américas, destacando las contribuciones, los desafíos y las perspectivas para fortalecer las acciones de salud pública.

**Métodos:**

este estudio teórico-reflexivo se realizó entre marzo y abril de 2025, con base en documentos oficiales de la Organización Panamericana de la Salud, especialmente las versiones de 2002 y 2021 de las funciones esenciales. Los materiales se identificaron mediante una búsqueda en el repositorio institucional de la Organización Panamericana de la Salud, incluyendo documentos normativos y descriptivos. El análisis se estructuró en cuatro etapas: evolución histórica; actualización conceptual de las funciones; impacto en la realidad brasileña; y contribuciones de enfermería.

**Resultados:**

las funciones esenciales actualizadas incorporaron los determinantes sociales. La enfermería destaca por su rol estratégico en la vigilancia, la promoción de la salud y la formulación de políticas públicas.

**Consideraciones Finales:**

las funciones esenciales actualizadas amplían las oportunidades de la enfermería para reducir las desigualdades y fortalecer sistemas de salud resilientes e inclusivos.

## INTRODUCTION

The Essential Public Health Functions (EPHF) initiative was launched in the 1980s by the Institute of Medicine, now known as the National Academy of Medicine, in the United States, as a response to the decline of public health in the United States. At that time, three primary functions were established: health policy analysis; policy development; and service delivery assurance. These functions play a crucial role in ensuring that governments implement effective, high-quality public health programs^(1)^.

In response to these guidelines, several initiatives emerged at the national, regional, and global levels. In 1994, the U.S. Centers for Disease Control and Prevention identified ten fundamental public healthcare services, while the World Health Organization conducted a Delphi survey that resulted in the first global list of EPHF in 1997. The goal was to create an international consensus on public health priorities and ensure the provision of minimum services, especially in developing nations^(2)^.

It is important to note that in the 1980s and 1990s, many countries in the Americas began implementing reforms to their health systems with the aim of promoting greater equity, access, and efficiency. These transformations focused on modifying the structure and organization of healthcare services. Although essential to consolidating public health as a social and institutional responsibility, strengthening leadership in healthcare institutions has not always received due attention, especially in contexts of high demand for government responses^(3)^.

As a result, Pan American Health Organization (PAHO) members began to recognize that healthcare professionals represent the essential foundation for the implementation and development of health projects, actions, and services for the population. Thus, in the early 2000s, PAHO encouraged the creation of a document for all countries in the Americas that defined EPHF through guidelines, proposing a new model of interaction between institutional and social structures, and highlighting the responsibilities in its implementation. This highlighted not only services and programs, but also healthcare professionals’ competences, their interrelationships, systems, and values^(4)^.

Therefore, to address emerging challenges and ensure the effective functioning of the health system, PAHO member nations began developing a public health platform model in 2000 to strengthen public health leadership in the Americas. In 2002, PAHO launched the first version of this model titled “*La Salud Pública en las Américas: Nuevos Conceptos, Análisis del Desempeño y Bases para la Acción*”. This initiative has driven the development of diagnostics and plans to strengthen health systems in countries such as Costa Rica, Cuba, and others in Central America and the Caribbean^(5)^.

The 2002 EPHF model also fostered collaborative actions, such as meetings of Central American health ministers and regional organizations like the Andean Community and the Southern Common Market, fostering cooperation in health and education policies. Over time, the EPHF approach was adapted to the specific needs of each country. By 2017, nations like Argentina, Chile, and Panama had implemented actions focused on their specificities, aiming to improve EPHF through training programs^(1)^.

It is worth mentioning that this document also had a significant impact in the Brazilian context, given the adaptation of EPHF to the Brazilian Health System (In Portuguese, *Sistema Único de Saúde* - SUS) decentralized management. The Brazilian National Council of Health Departments, in partnership with PAHO, developed methodologies to assess EPHF’s performance in Brazilian states, aiming to improve SUS state management^(4)^. This Brazilian experience, aligned with global guidelines, reinforced how EPHF were essential instruments for strengthening health systems.

EPHF review was also driven by public health challenges, such as the emergence of new infectious diseases. The crisis caused by the COVID-19 pandemic highlighted deficiencies and the pressing need for significant improvements in public health on a global scale, reaffirming ideas put forward in “*La Salud Pública en las Américas: Nuevos Conceptos, Análisis del Desempeño y Bases para la Acción*” in relation to past crises, such as the H1N1 epidemic, the Ebola outbreak, and the Chikungunya and Zika epidemics. These emergencies reiterated the importance of EPHF in ensuring access to quality health interventions^(2)^.

Nursing’s central and strategic role within this context stands out. Due to their proximity to the community and multidisciplinary work, nurses directly contribute to EPHF implementation and maintenance, being indispensable in responding to public health crises^(6)^. In practice, they are involved in critical areas such as health surveillance, public policy formulation, and social mobilization, acting as a link between healthcare services and the population. This role promotes equitable access and ensures quality interventions. Furthermore, the education and continuing training of these professionals are fundamental elements for addressing emerging challenges, such as disease outbreaks and social inequalities in health^(7)^.

Thus, PAHO documents consolidate nursing as a key force in achieving EPHF objectives. Nurses not only provide direct care but also strengthen the institutional capacities needed for public health, contributing to more robust and resilient health systems.

## OBJECTIVES

To reflect on nursing’s role in EPHF in the Americas, highlighting its contributions, challenges, and opportunities to strengthen public health actions in the region, emphasizing the importance of continuous capacity development to face emerging challenges in the health scenario.

## METHODS

This is a theoretical-reflective study based on documentary analysis. The search was conducted between March and April 2025 through direct access to the PAHO institutional repository (https://iris.paho.org). The terms used as main descriptors were “*Funções Essenciais de Saúde Pública*”, “Essential Public Health Functions” and “*Funções Essenciais de Saúde Pública*”, filtered by language (Portuguese, Spanish, and English) and date (2000 to 2025).

Documents with a normative or descriptive scope on EPHF in the Americas were included, prioritizing official PAHO materials, as well as scientific publications that addressed reflections, assessments, or practical applications of EPHF with an emphasis on nursing practice. Duplicate documents, journalistic reports, and content without institutional or scientific support were excluded.

Analysis was organized into four stages, defined *a priori* based on the study objectives: (1) the historical trajectory of EPHF and nursing recognition in the Americas; (2) advances and new perspectives of EPHF with the 2021 update; (3) the impact of EPHF on the Brazilian reality and nursing contribution; and (4) contributions, challenges, and future perspectives of nursing in EPHF in the Americas. The reading of the documents followed an interpretative approach, with an emphasis on identifying meanings and arguments related to nursing’s role and the structuring of public health in the countries of the Americas.

## RESULTS

### The historical trajectory of essential public health functions and nursing recognition in the Americas

EPHF evolution in the Americas represents an ongoing response to the public health challenges faced in the region. This movement began in the 1980s, driven by the need to establish both structural and operational functions to enhance the responsiveness of public health systems. In 2000, PAHO member countries decided to create a theoretical and practical framework that resulted, in 2002, in the release of the first document on EPHF entitled “*La Salud Pública en las Américas: Nuevos Conceptos, Análisis del Desempeño y Bases para la Acción*”^(1)^.

This report was groundbreaking in recognizing and characterizing EPHF, enabling Latin American and Caribbean nations to conduct institutional diagnoses and develop strategies to improve their public health systems. The proposal not only facilitated policy adoption at the national and regional levels but also encouraged cooperation among countries, reinforcing public health as a fundamental responsibility of governments^(2)^.

The documents on EPHF in the Americas, both the 2002 and 2021 editions, emphasize nursing’s fundamental role in carrying out these functions. The documents highlight the crucial importance of human resources, including nursing professionals, for the effective implementation of EPHF, especially in surveillance activities, public policy promotion, and social engagement^(1,2)^.

The 2002 edition already highlighted the importance of strengthening healthcare workers’ role, including nurses, as key elements in public health. This understanding is expanded in the 2021 report, which emphasizes the need to develop skills and provide continuing education for professionals in the sector. Nursing, due to its diverse role and close connection with the community, is considered essential for implementing integrated and intersectoral actions related to the social factors that influence health, promoting access to and quality of healthcare services^(2)^.

### Advances and new perspectives on essential public health functions with the 2021 update

The 2002 Public Health in the Americas initiative achieved significant results, particularly in mobilizing diverse stakeholders to discuss the issue and in advancing EPHF measurement, although there was a tendency to associate EPHF solely with its measurement. This association did not sufficiently explore its role as a tool for strengthening health authorities and its impact on health system change^(6)^.

The EPHF 2021 review, through the document “The Essential Public Health Functions in the Americas - A Renewal for the 21^st^ Century. Conceptual Framework and Description”, demonstrated the urgency of aligning the guidelines with new demands, including social factors that influence health and the effects of global crises, such as the COVID-19 pandemic. The proposed review aims not only to safeguard EPHF as a cornerstone of public health, but also to expand their relevance and application in the face of current challenges, encouraging a more integrated perspective on public health in the Americas^(3)^.

In the Americas, approximately 30% of the population, equivalent to 279 million individuals, do not seek medical care when needed due to barriers that hinder access to adequate healthcare services and care. This lack of access results in approximately 1.7 million deaths annually in the region^(6)^.

PAHO highlights the intensification of contagious diseases such as measles and tuberculosis, the rise in sexually transmitted infections, and antimicrobial resistance. It also warns of the emergence of new health threats, such as COVID-19, Ebola, West Nile virus, and Zika. At the same time, population aging and changes in epidemiological and socioeconomic conditions have increased the prevalence of chronic noncommunicable diseases, mental disorders, disabilities, traffic accidents, and domestic and interpersonal violence^(6)^.

The 11 EPHF are focused on analyzing the population’s health status and the factors that compromise it. These functions include developing guidelines to improve these conditions, addressing the social determinants of health, ensuring appropriate resource allocation, and promoting universal access to public health interventions and services^(3)^.

These functions are conceived as fundamental institutional capabilities that countries must strengthen to ensure effective public health action. Furthermore, civil society’s and other strategic actors’ participation is crucial not only in policy implementation but also in their development and assessment. Intersectoral collaboration to address the social determinants of health has been gaining relevance and consolidating itself as an essential part of advancing public health^(4)^.

The EPHF 2021 update reflects an adaptation to contemporary challenges, including pandemics, persistent inequalities, and socioeconomic crises. Notable advances include the incorporation of a human rights-centered approach, an emphasis on universal health, and a strengthening of health systems’ capacity to respond to emergencies. These transformations position EPHF as a crucial point for the development and implementation of policies that ensure more just and sustainable public health^(2)^.

Looking ahead, the integration of new technologies, such as artificial intelligence and advanced information systems, promises to optimize health condition assessment and resource allocation. Additionally, a commitment to strengthening participatory and intersectoral governance could accelerate progress toward more resilient and inclusive health systems. Thus, the renewed EPHF represent a guide not only for addressing current challenges but also for preparing health systems to respond to future crises more efficiently and humanely^(3)^.

### The impact of essential public health functions on the Brazilian reality and nursing contribution

EPHF, updated by PAHO in 2021, provides a strategic guide for improving health systems in the Americas, with an emphasis on Brazil. In a nation marked by social and regional inequalities, EPHF play a fundamental role in ensuring that health policies effectively respond to the population’s needs. Combining approaches that consider the social determinants of health is especially relevant in Brazil, where factors such as poverty, low educational levels, and unequal access to basic sanitation services directly affect health indicators. Thus, EPHF implementation by health managers can facilitate greater equity and effectiveness in services, in line with SUS principles, which provide for universality and comprehensiveness^(3)^.

In Brazil, nursing’s role is fundamental to EPHF implementation, as this category represents the largest healthcare workforce in the country, working directly in primary care, hospitals, and healthcare service administration. Nursing professionals play vital roles in epidemiological surveillance, health education, promoting healthy habits, and responding to public health emergencies. Furthermore, nurses’ ability to implement programs such as the Family Health Strategy and monitor vulnerable populations emphasizes their contribution to achieving the objectives proposed by EPHF, such as expanding access and reducing inequalities^(3)^.

The fragmentation of healthcare systems poses a structural challenge to EPHF implementation, especially in countries with federal realities and poorly integrated care networks like Brazil. This disconnect between levels of care compromises the continuity of care and the coordinated implementation of public policies. In this context, nursing training should prioritize competences focused on care management, intersectoral coordination, and community leadership, empowering professionals to act as integrators among services and as key players in the reorganization of healthcare networks^(7)^.

An important aspect of EPHF in Brazil is the emphasis on community participation and collaboration between sectors that are already part of SUS primary care. Nursing professionals, due to their close proximity to communities, play a crucial role in encouraging this participation and involving citizens in the planning and supervision of health initiatives. Through actions such as vaccination campaigns, disease prevention, and mental health promotion, nurses contribute to creating an environment that fosters mobilization and social control, in line with EPHF guidelines, reinforcing society’s role in public policies^(6,7)^.

The vision of technological and digital advancement, highlighted in EPHF, offers a valuable opportunity for the nursing profession in Brazil. The use of health information systems, such as e-SUS, along with the adoption of telemonitoring technologies, enables nurses to remotely monitor patients, improve care, and monitor the progression of chronic diseases. Nurses’ participation in this digital environment, supported by continuing training and access to appropriate tools, is essential to ensure the inclusive and effective implementation of technologies, expanding the impact of public health initiatives^(8)^.

In short, nursing’s role in strengthening EPHF in Brazil is deeply linked to its leadership role in interdisciplinary teams and its ability to manage various spheres of the healthcare system. Overcoming the challenges faced in the country, such as reducing regional inequalities and improving quality of care, requires that nursing professionals be prepared to serve as a link between managers, communities, and other healthcare team members. Thus, by integrating EPHF principles into their activities, nurses not only promote the implementation of more efficient policies but also contribute to the development of a more just, resilient healthcare system that is better equipped to face the challenges of the 21^st^ century^(9)^.

### Contributions, challenges, and future perspectives of nursing in essential public health functions in the Americas

Nursing’s contributions to public health programs in the Americas are broad and fundamental, given that nurses play a vital role in various dimensions of public health. Their work in health promotion, disease prevention, and direct patient care positions nursing as a key force in strengthening EPHF. Furthermore, nurses are indispensable in implementing public policies, epidemiological surveillance, and caring for vulnerable groups, especially in areas marked by inequalities, such as many parts of the Americas. Because of their ability to work in interdisciplinary teams, in communities, through the exchange of knowledge and mutual learning, and as leaders in health initiatives, nursing is an essential profession for the advancement of public health^(3)^.

Despite having made important contributions, nursing faces barriers that compromise its effective participation in strengthening EPHF. Among the main challenges are the scarcity of resources, the high workload, and the devaluation of the profession in various areas of the region.

EPHF implementation varies among countries in the Americas, reflecting their political structures, health systems, and training strategies. In Cuba, for instance, there is a strong integration between teaching and practice in public health, with nursing playing a strategic role in health surveillance and promotion. In Argentina and Chile, federal initiatives for EPHF training and institutional performance assessment stand out. Mexico has made progress in incorporating EPHF into primary care programs, but still faces challenges related to system fragmentation and the valorization of nursing at the community level. This diversity highlights the need to adapt nursing practices to local contexts, ensuring critical training, political participation, and intersectoral action as foundations for strengthening EPHF in the Americas^(1)^.

Furthermore, disparities in training and access to continuing education opportunities among countries in the Americas hinder the development of advanced public health skills. These issues limit nursing’s ability to lead and innovate in EPHF implementation, especially in a context where the demand for integrated and technological actions is increasing^(5)^.

Expectations for the future are encouraging, particularly with the growing appreciation for nursing’s crucial role in times of health crises, such as the COVID-19 pandemic. This recognition has led to the creation of initiatives aimed at improving nursing professionals’ education and training in public health. The implementation of interprofessional training programs, the greater adoption of digital technologies, and the adoption of policies that ensure better working conditions are some of the strategies that can enhance nursing performance in EPHF. Furthermore, strengthening nurses’ role as leaders in public health can foster greater participation of the profession in policy creation and implementation.

Ultimately, the future of nursing in public healthcare institutions is tied to its ability to increase its presence in policy-making and management bodies in public health. Promoting nursing research, especially in the public health sector, can help generate evidence to support more innovative and effective actions. Nursing has the potential to change the health landscape in the Americas, acting as a link between health managers and communities’ concrete needs, ensuring that public healthcare institutions are implemented in a fair, efficient, and sustainable manner. Therefore, investing in the valorization and strengthening of nursing is crucial so that public healthcare institutions can fully achieve their potential for the benefit of the populations of the Americas^(10)^.

## FINAL CONSIDERATIONS

EPHF are a crucial step in strengthening health networks in the Americas, offering an integrated model to address current challenges and promote health equitably. EPHF evolution, which began with their first proposal in 2002 and was revised in 2021, illustrates an adaptation to the new demands brought about by the social determinants of health, global crises, and economic and social changes. In this context, nursing stands out as a vital component, playing fundamental roles in health surveillance, prevention, and promotion, as well as serving as a connector between communities, administrators, and other healthcare professionals.

In Brazil and several other countries in the Americas, the nursing profession has played a crucial role in EPHF implementation, especially in programs focused on primary care and reducing social inequalities. However, challenges remain, such as the need for greater recognition of the profession, improvements in working conditions, and access to continuing education. Overcoming these obstacles is essential to harnessing the full potential of nursing professionals as leaders in the management and implementation of public health policies. Furthermore, it is vital that nurses assume leadership and participation positions, expanding their influence in the planning and assessment phases of EPHF.

The prospects for the future of nursing in public healthcare institutions are encouraging, particularly with the advent of digital technologies, which have the potential to expand the reach of public health initiatives and improve processes. The adoption of these tools, combined with more collaborative governance and the strengthening of interprofessional training, will allow nursing to maintain its prominent position in the transformation of health systems. Prioritizing nurses’ appreciation and development not only increases the effectiveness of public healthcare institutions but also helps create more inclusive, resilient health systems that are better equipped to face the challenges of the 21^st^ century.

Since this is a theoretical-reflective study, the findings are based on a critical interpretation of PAHO documents, which may limit the generalizability of results. The lack of empirical data also limits the understanding of concrete nursing practices in different contexts in the Americas.

## Data Availability

The research data are available within the article.
