# Supplementary material for: Nursing and the essential public health functions framework in the Americas: reflections and perspectives
Source: Rev Bras Enferm. 2026 Jan 26;78(6):e20250121. doi: 10.1590/0034-7167-2025-0121 (PMC12854264; doi:10.1590/0034-7167-2025-0121)
Supplement: Translation

# Translation: Enfermagem e o referencial das funções essenciais de saúde pública nas Américas: reflexões e perspectivas

**DOI:** 10.1590/0034-7167-2025-0121pt

**Authors:** Eduardo Neves da Cruz de Souza, Maria de Lourdes de Almeida, Juana Elvira Suárez Conejero, Aida Maris Peres

**Affiliations:** I Universidade Federal do Paraná. Curitiba, Paraná, Brasil; II Universidade Estadual do Oeste do Paraná. Foz do Iguaçu, Paraná, Brasil; III Universidad Nacional Autónoma de México. Ciudad de México, México

**Keywords:** Enfermagem, Prática Profissional, Competência Profissional, Funções Essenciais da Saúde Pública, Américas.

## Abstract

**Objetivos:**

refletir sobre o papel da enfermagem nas funções essenciais de saúde pública nas Américas, destacando contribuições, desafios e perspectivas no fortalecimento das ações de saúde pública.

**Métodos:**

estudo teórico-reflexivo, realizado entre março e abril de 2025, com base em documentos oficiais da Organização Pan-Americana da Saúde, especialmente as versões de 2002 e 2021 das funções essenciais. Os materiais foram identificados por busca no repositório institucional da Organização Pan-Americana da Saúde, incluindo documentos normativos e descritivos. A análise foi estruturada em quatro etapas: evolução histórica; atualização conceitual das funções; impacto na realidade brasileira; e contribuições da enfermagem.

**Resultados:**

atualização das funções essenciais incorporou determinantes sociais. A enfermagem se destaca por seu papel estratégico na vigilância, promoção da saúde e formulação de políticas públicas.

**Considerações Finais:**

as funções essenciais atualizadas ampliam oportunidades para atuação da enfermagem na redução de desigualdades e fortalecimento de sistemas de saúde resilientes e inclusivos.

## INTRODUÇÃO

A iniciativa Funções Essenciais de Saúde Pública (FESP) teve início na década de 80 pelo *Institute of Medicine*, hoje conhecido como *National Academy of Medicine*, nos Estados Unidos, como uma reação ao declínio da saúde pública no território norte-americano. Naquela época, três funções primordiais foram estabelecidas: análise das políticas de saúde; desenvolvimento de políticas; e certeza da oferta de serviços. Essas funções desempenham um papel crucial para garantir que os governos implementem programas de saúde pública eficazes e de alta qualidade^(1)^.

Atendendo a essas orientações, múltiplas iniciativas emergiram em níveis nacional, regional e global. Em 1994, os Centros de Controle e Prevenção de Doenças dos Estados Unidos identificaram dez serviços fundamentais de saúde pública, enquanto a Organização Mundial da Saúde conduziu uma pesquisa Delphi que resultou na primeira lista mundial de FESP em 1997. A meta era criar um consenso internacional sobre as prioridades em saúde pública e assegurar a oferta mínima de serviços, especialmente em nações em desenvolvimento^(2)^.

É importante destacar que, nas décadas de 1980 e 1990, muitos países das Américas começaram a implementar reformas em seus sistemas de saúde com o objetivo de promover maior equidade, acesso e eficiência. Essas transformações se concentraram em modificar a estrutura e a organização dos serviços de saúde. Apesar de ser fundamental para consolidar a saúde pública como responsabilidade social e institucional, o fortalecimento da liderança nas instituições de saúde nem sempre recebeu a devida atenção, sobretudo em contextos de elevada demanda por respostas governamentais^(3)^.

Com isso, os membros da Organização Pan-Americana da Saúde (OPAS) passaram a reconhecer que os profissionais de saúde representam a fundamentação essencial para realização e o desenvolvimento de projetos, ações e serviços de saúde destinados à população. Assim, no início dos anos 2000, a OPAS incentivou a criação de um documento para todos os países das Américas que definisse as FESP por meio de diretrizes, propondo um novo modelo de interação entre as estruturas institucionais e sociais, e ressaltando as responsabilidades em sua implementação. Isso destacou não apenas os serviços e programas, mas também as competências dos profissionais de saúde, suas inter-relações, sistemas e valores^(4)^.

A partir disso, com o objetivo de lidar com os desafios que surgem e assegurar um funcionamento eficaz do sistema de saúde, as nações que fazem parte da OPAS começaram, em 2000, o desenvolvimento de um modelo de FESP destinado a reforçar a liderança na área da saúde pública nas Américas. Em 2002, a OPAS lançou a primeira versão deste modelo intitulada “*La Salud Pública en las Américas: Nuevos Conceptos, Análisis del Desempeño y Bases para la Acción*”. Essa iniciativa impulsionou o desenvolvimento de diagnósticos e planos para fortalecer sistemas de saúde em países como Costa Rica, Cuba, e outros da América Central e Caribe^(5)^.

O modelo FESP de 2002 também fomentou ações colaborativas, como as reuniões de ministros da saúde da América Central e de organizações regionais como a Comunidade Andina e o Mercado Comum do Sul, promovendo a cooperação em políticas de saúde e educação. Com o tempo, a abordagem das FESP foi adaptada às necessidades específicas de cada país. Até 2017, nações como Argentina, Chile e Panamá implementaram ações focadas nas suas peculiaridades, visando aprimorar as FESP por meio de programas de capacitação^(1)^.

Vale mencionar que este documento também obteve impacto significativo no contexto brasileiro, diante da adaptação das FESP à gestão descentralizada do Sistema Único de Saúde (SUS), na medida em que o Conselho Nacional de Secretários de Saúde, em parceria com a OPAS, desenvolveu metodologias para avaliar o desempenho das FESP nos estados brasileiros, visando aprimorar a gestão estadual do SUS^(4)^. Essa experiência brasileira, alinhada às diretrizes globais, reforçou como as FESP foram instrumentos essenciais para fortalecer os sistemas de saúde.

A revisão das FESP também foi impulsionada pelos desafios de saúde pública, como o surgimento de novas doenças infecciosas. A crise provocada pela pandemia de COVID-19 evidenciou deficiências e a necessidade premente de melhorias significativas na saúde pública em escala global, reafirmando ideias apresentadas em “*La Salud Pública en las Américas: Nuevos Conceptos, Análisis del Desempeño y Bases para la Acción*” em relação a crises passadas, como a epidemia de H1N1, o surto de ebola, além das epidemias de Chikungunya e Zika. Essas emergências reiteraram a importância das FESP para assegurar o acesso a intervenções de saúde de qualidade^(2)^.

Destaca-se o papel central e estratégico da enfermagem dentro desse contexto. Por sua proximidade com a comunidade e atuação multidisciplinar, os enfermeiros contribuem diretamente para implementação e sustentação das FESP, sendo indispensáveis nas respostas a crises de saúde pública^(6)^. Na prática, estão envolvidos em áreas críticas como vigilância em saúde, formulação de políticas públicas e mobilização social, funcionando como elo entre os serviços de saúde e a população. Essa atuação promove o acesso equitativo e garantia de intervenções de qualidade. Além disso, a educação e a capacitação contínua desses profissionais são elementos fundamentais para o enfrentamento de desafios emergentes, como surtos de doenças e desigualdades sociais em saúde^(7)^.

Dessa forma, os documentos da OPAS consolidam a enfermagem como uma força essencial para alcançar os objetivos das FESP. Os enfermeiros não apenas oferecem cuidados diretos, mas também fortalecem as capacidades institucionais necessárias para a saúde pública, contribuindo para sistemas de saúde mais robustos e resilientes.

## OBJETIVOS

Refletir sobre o papel da enfermagem nas FESP nas Américas, destacando suas contribuições, desafios e oportunidades para fortalecer as ações de saúde pública na região, ressaltando a importância do desenvolvimento contínuo de capacidades para enfrentar os desafios emergentes no cenário de saúde.

## MÉTODOS

Trata-se de estudo teórico-reflexivo baseado em análise documental. A busca foi realizada entre março e abril de 2025 por meio de acesso direto ao repositório institucional da OPAS (https://iris.paho.org). Foram utilizados como descritores principais os termos “Funções Essenciais de Saúde Pública”, “*Essential Public Health Functions*” e “Funções Essenciais de Saúde Pública”, com filtragem por idioma (português, espanhol e inglês) e por data (de 2000 a 2025).

Foram incluídos documentos com escopo normativo ou descritivo sobre as FESP nas Américas, priorizando materiais oficiais da OPAS, bem como publicações científicas que abordassem reflexões, avaliações ou aplicações práticas das FESP com ênfase na atuação da enfermagem. Excluíram-se documentos duplicados, informes jornalísticos e conteúdos sem respaldo institucional ou científico.

A análise foi organizada em quatro etapas, definidas, *a priori*, com base nos objetivos do estudo: (1) a trajetória histórica das FESP e reconhecimento da enfermagem nas Américas; (2) avanços e novas perspectivas das FESP com a atualização de 2021; (3) o impacto das FESP na realidade brasileira e contribuição da enfermagem; e (4) contribuições, desafios e perspectivas futuras da enfermagem nas FESP nas Américas. A leitura dos documentos seguiu abordagem interpretativa, com ênfase na identificação de sentidos e argumentos relacionados à atuação da enfermagem e à estruturação da saúde pública nos países das Américas.

## RESULTADOS

### A trajetória histórica das funções essenciais de saúde pública e o reconhecimento da enfermagem nas Américas

A evolução das FESP nas Américas representa uma resposta contínua às dificuldades de saúde pública enfrentadas na região. Este movimento começou na década de 1980, motivado pela necessidade de estabelecer funções tanto estruturais quanto operacionais para aprimorar a capacidade de reação dos sistemas de saúde pública. Em 2000, os países membros da OPAS decidiram criar um referencial teórico e prático que resultou, em 2002, no lançamento do primeiro documento sobre as FESP intitulado “*La Salud Pública en las Américas: Nuevos Conceptos, Análisis del Desempeño y Bases para la Acción*”^(1)^.

Este relatório foi inovador ao reconhecer e caracterizar as FESP, possibilitando que nações da América Latina e do Caribe conduzissem diagnósticos institucionais e elaborassem estratégias para aprimorar seus sistemas de saúde pública. A proposta não apenas facilitou a adoção de políticas a nível nacional e regional, mas também estimulou a cooperação entre os países, reforçando a saúde pública como uma responsabilidade fundamental dos governos^(2)^.

Os documentos sobre as FESP nas Américas, tanto na edição de 2002 quanto na de 2021, enfatizam o papel fundamental da enfermagem na realização dessas funções. Os documentos ressaltam a relevância determinante dos recursos humanos, incluindo profissionais de enfermagem, para a efetiva execução das FESP, especialmente em atividades de vigilância, promoção de políticas públicas e engajamento social^(1,2)^.

A edição de 2002 já apontava a importância de reforçar a atuação dos trabalhadores da saúde, incluindo os enfermeiros, como elementos-chave na saúde pública. Esse entendimento é expandido no relatório de 2021, que ressalta a necessidade de desenvolver habilidades e educação continuada para os profissionais do setor. A enfermagem, pela sua atuação diversificada e estreita ligação com a comunidade, é considerada essencial para implementar ações integradas e intersetoriais referentes aos fatores sociais que influenciam a saúde, promovendo o acesso e a qualidade dos serviços de saúde^(2)^.

### Avanços e novas perspectivas das funções essenciais de saúde pública com a atualização de 2021

A iniciativa Saúde Pública nas Américas de 2002 alcançou resultados significativos, especialmente na mobilização de diferentes partes interessadas para discutir o assunto e nos avanços na medição das FESP, apesar de ter sido observada uma tendência de associar as FESP apenas à sua mensuração. Essa associação não explorou suficientemente a sua função como instrumento de fortalecimento das autoridades de saúde e o seu efeito na mudança dos sistemas de saúde^(6)^.

A revisão das FESP em 2021, por meio do documento “As Funções Essenciais de Saúde Pública nas Américas - Uma Renovação para o Século 21. Marco conceitual e descrição”, demonstrou a urgência de alinhar as orientações às novas demandas, incluindo fatores sociais que influenciam a saúde e os efeitos de crises globais, como a pandemia de COVID-19. A proposta apresentada visa não apenas resguardar as FESP enquanto alicerces da saúde pública, mas também ampliar sua relevância e aplicação diante dos desafios atuais, incentivando uma perspectiva mais integrada da saúde pública nas Américas^(3)^.

Nas Américas, aproximadamente 30% da população, o que equivale a 279 milhões de indivíduos, não busca assistência médica quando necessário em razão de barreiras dificultadoras do acesso a serviços e cuidados de saúde adequados. Essa falta de acesso resulta em cerca de 1,7 milhão de mortes anuais na região^(6)^.

A OPAS destaca a intensificação de doenças contagiosas como sarampo e tuberculose, o aumento de infecções sexualmente transmissíveis e a resistência antimicrobiana. Alerta-se ainda para o surgimento de novas ameaças à saúde, como COVID-19, ebola, vírus do Nilo Ocidental e Zika. Paralelamente, o envelhecimento populacional e as mudanças nas condições epidemiológicas e socioeconômicas têm elevado a prevalência de doenças crônicas não transmissíveis, transtornos mentais, incapacidades, acidentes de trânsito, e violência doméstica e interpessoal^(6)^.

As 11 FESP são orientadas para análise da condição de saúde da população e dos fatores que comprometem esse estado. Essas funções incluem o desenvolvimento de diretrizes para melhorar essas condições, a abordagem dos determinantes sociais da saúde, a alocação adequada de recursos e a promoção do acesso universal a intervenções e serviços de saúde pública^(3)^.

Essas funções são concebidas como capacidades institucionais fundamentais que os países devem fortalecer para garantir uma atuação eficaz em saúde pública. Além disso, a participação da sociedade civil e de outros atores estratégicos é crucial não apenas na implementação de políticas, mas também no desenvolvimento e avaliação das mesmas. A colaboração intersetorial para combater os determinantes sociais da saúde vem ganhando relevância e se consolidando como parte essencial do avanço da saúde pública^(4)^.

A atualização das FESP em 2021 reflete uma adaptação aos desafios contemporâneos, incluindo pandemias, desigualdades persistentes e crises socioeconômicas. Entre os avanços notáveis, estão a incorporação de uma abordagem centrada nos direitos humanos, a ênfase na saúde universal e o fortalecimento da capacidade de resposta dos sistemas de saúde a emergências. Essas transformações colocam as FESP como ponto crucial para o desenvolvimento e execução de políticas que assegurem uma saúde pública mais justa e sustentável^(2)^.

No horizonte das perspectivas, a integração de novas tecnologias, como inteligência artificial e sistemas de informação avançados, promete otimizar a avaliação das condições de saúde e a alocação de recursos. Adicionalmente, o compromisso de fortalecer a governança participativa e intersetorial poderá acelerar os progressos rumo a sistemas de saúde mais resilientes e inclusivos. Assim, as FESP renovadas representam um guia não apenas para enfrentar os desafios atuais, mas também para preparar os sistemas de saúde para responder a crises futuras de maneira mais eficiente e humana^(3)^.

### O impacto das funções essenciais de saúde pública na realidade brasileira e contribuição da enfermagem

As FESP, atualizadas pela OPAS em 2021, fornecem um guia estratégico para o aprimoramento dos sistemas de saúde nas Américas, com ênfase no Brasil. Em uma nação marcada por desigualdades sociais e regionais, as FESP desempenham um papel fundamental para garantir que as políticas de saúde respondam de forma eficaz às necessidades da população. A combinação de abordagens que considerem os determinantes sociais da saúde é especialmente pertinente no Brasil, onde aspectos como pobreza, baixo nível educacional e acesso desigual a serviços de saneamento básico afetam os indicadores de saúde de forma direta. Dessa forma, a implementação das FESP pelos gestores de saúde pode facilitar uma maior equidade e eficácia nos serviços, em consonância com os princípios do SUS, que preveem a universalidade e a integralidade^(3)^.

No Brasil, o papel da enfermagem é fundamental para efetivação das FESP, uma vez que essa categoria representa a maior força de trabalho na área da saúde no país, atuando de maneira direta na atenção primária, em hospitais e na administração de serviços de saúde. Os profissionais de enfermagem exercem funções vitais na vigilância epidemiológica, educação em saúde, promoção de hábitos saudáveis e resposta a situações de emergência em saúde pública. Ademais, a habilidade dos enfermeiros em implementar programas como a Estratégia Saúde da Família e em monitorar populações em situação de vulnerabilidade acentua sua contribuição para atingir os objetivos propostos pelas FESP, como a ampliação do acesso e a diminuição das desigualdades^(3)^.

A fragmentação dos sistemas de saúde representa um desafio estrutural à efetivação das FESP, especialmente em países com realidades federativas e redes de atenção ainda pouco integradas como o Brasil. Essa desarticulação entre os níveis de atenção compromete a continuidade do cuidado e a implementação de políticas públicas de forma coordenada. Nesse contexto, a formação em enfermagem deve priorizar competências voltadas à gestão do cuidado, articulação intersetorial e liderança comunitária, capacitando os profissionais para atuar como elementos de integração entre os serviços e como protagonistas da reorganização das redes de atenção à saúde^(7)^.

Um ponto importante das FESP no Brasil é a valorização da participação da comunidade e da colaboração entre setores que já fazem parte da atenção básica do SUS. Os profissionais de enfermagem, pela sua proximidade com as comunidades, possuem um papel crucial no incentivo dessa participação e envolvimento dos cidadãos no planejamento e na supervisão das iniciativas de saúde. Através de ações como campanhas de vacinação, prevenção de doenças e promoção da saúde mental, os enfermeiros contribuem para formação de um ambiente que favorece a mobilização e o controle social, em consonância com as orientações das FESP, reforçando a atuação da sociedade nas políticas públicas^(6,7)^.

A visão de avanço tecnológico e digital, ressaltada nas FESP, oferece uma chance valiosa para a profissão de enfermagem no Brasil. A utilização de sistemas de informação em saúde, como o e-SUS, junto com a adoção de tecnologias de telemonitoramento, possibilita que os enfermeiros realizem o acompanhamento remoto de pacientes, melhorem o atendimento e supervisem a progressão de enfermidades crônicas. A participação dos enfermeiros nesse ambiente digital, apoiada por capacitação contínua e acesso a ferramentas apropriadas, é essencial para assegurar a implementação inclusiva e efetiva das tecnologias, ampliando o impacto das iniciativas de saúde pública^(8)^.

Em suma, a atuação da enfermagem no fortalecimento das FESP no Brasil está profundamente relacionada à sua função de liderança em equipes interdisciplinares e à sua habilidade de gerenciar em diversas esferas do sistema de saúde. A superação dos desafios enfrentados no país, como a diminuição das desigualdades regionais e a melhoria da qualidade no atendimento, demanda que os profissionais de enfermagem estejam preparados para servir como um conectivo entre gestores, comunidades e outros integrantes da equipe de saúde. Dessa forma, ao integrar os princípios das FESP em suas atividades, os enfermeiros não apenas favorecem a execução de políticas mais eficientes, mas também colaboram para a formação de um sistema de saúde mais justo, resiliente e apto a enfrentar os desafios do século XXI^(9)^.

### Contribuições, desafios e perspectivas futuras da enfermagem nas funções essenciais de saúde pública nas Américas

As contribuições da enfermagem para as FESP nas Américas são amplas e fundamentais, dado que os enfermeiros exercem um papel vital em várias dimensões da saúde pública. Seu trabalho na promoção da saúde, na prevenção de enfermidades e no atendimento direto aos pacientes posiciona a enfermagem como uma das principais forças no fortalecimento das FESP. Ademais, os enfermeiros são indispensáveis na execução de políticas públicas, na vigilância epidemiológica e no cuidado de grupos vulneráveis, especialmente em áreas marcadas por desigualdades, como muitas partes das Américas. Por suas habilidades de atuação em equipes interdisciplinares, em comunidades, através da troca de saberes e aprendizado mútuo, e de liderança em iniciativas de saúde, a enfermagem é uma profissão essencial para o progresso da saúde pública^(3)^.

Embora tenha feito contribuições importantes, a enfermagem enfrenta barreiras que comprometem sua participação efetiva no fortalecimento das FESP. Entre os principais desafios, destacam-se a escassez de recursos, a alta carga de trabalho e a desvalorização da profissão em diversas áreas da região.

A implementação das FESP varia entre os países das Américas, refletindo suas estruturas políticas, sistemas de saúde e estratégias de formação. Em Cuba, por exemplo, observa-se uma forte integração entre ensino e prática em saúde pública, com atuação estratégica da enfermagem na vigilância e promoção da saúde. Já na Argentina e no Chile, destacam-se iniciativas federais para capacitação em FESP e avaliação do desempenho institucional. O México avançou na incorporação das FESP em programas de atenção primária, mas ainda enfrenta desafios relacionados à fragmentação do sistema e à valorização da enfermagem em nível comunitário. Essa diversidade evidencia a necessidade de adaptar a atuação da enfermagem aos contextos locais, garantindo formação crítica, participação política e atuação intersetorial como bases para o fortalecimento das FESP nas Américas^(1)^.

Ademais, a disparidade na formação e no acesso a oportunidades de educação continuada entre os países das Américas prejudica o desenvolvimento de habilidades avançadas em saúde pública. Essas questões restringem a capacidade da enfermagem de liderar e inovar na implementação das FESP, principalmente em um contexto onde a demanda por ações integradas e tecnológicas está aumentando^(5)^.

As expectativas para o futuro são encorajadoras, principalmente com o aumento da valorização do papel fundamental da enfermagem em momentos de crise sanitária, como a pandemia de COVID-19. Esse reconhecimento tem levado à criação de iniciativas voltadas para aprimorar a educação e o treinamento dos profissionais de enfermagem na área da saúde pública. A implementação de programas de formação interprofissional, a maior adoção de tecnologias digitais e a adoção de políticas que assegurem melhores condições de trabalho são algumas das estratégias que podem potencializar a atuação da enfermagem nas FESP. Ademais, o fortalecimento da presença dos enfermeiros como líderes na saúde pública pode favorecer uma maior participação da categoria na criação e execução de políticas.

Em última análise, o futuro da enfermagem nas FESP está atrelado à habilidade de aumentar sua presença em instâncias de decisão política e gestão na área da saúde pública. Promover a pesquisa em enfermagem, especialmente no setor da saúde coletiva, pode ajudar na geração de evidências que fundamentem ações mais inovadoras e eficazes. A enfermagem possui a capacidade de mudar o panorama da saúde nas Américas, atuando como um elo entre os gestores de saúde e as necessidades concretas das comunidades, garantindo que as FESP sejam implementadas de forma justa, eficiente e sustentável. Assim, é fundamental investir na valorização e no fortalecimento da enfermagem para que as FESP possam atingir plenamente seu potencial em benefício das populações das Américas^(10)^.

## CONSIDERAÇÕES FINAIS

As FESP constituem um ponto crucial para o fortalecimento das redes de saúde nas Américas, oferecendo um modelo integrado para lidar com desafios atuais e promover a saúde de maneira justa. A evolução das FESP, que começou com sua primeira proposta em 2002, revisada em 2021, ilustra uma adaptação às novas exigências trazidas pelos determinantes sociais da saúde, crises globais, e mudanças econômicas e sociais. Nesse cenário, a enfermagem se destaca como uma componente vital, exercendo papéis fundamentais na vigilância, prevenção e promoção da saúde, além de servir como um conector entre as comunidades, administradores e outros profissionais de saúde.

No Brasil e em diversas nações americanas, a profissão de enfermagem tem desempenhado um papel crucial na implementação das FESP, especialmente em programas focados na atenção primária e na diminuição das desigualdades sociais. Entretanto, ainda existem desafios, como a necessidade de uma maior valorização da profissão, melhorias nas condições de trabalho e acesso a cursos de formação continuada. Ultrapassar esses obstáculos é fundamental para o aproveitamento de todo o potencial dos profissionais de enfermagem como líderes na gestão e execução de políticas públicas de saúde. Além disso, é vital que os enfermeiros assumam posições de liderança e participação, ampliando sua influência nas fases de planejamento e avaliação das FESP.

As perspectivas para o futuro da enfermagem nas FESP são animadoras, principalmente com a chegada de tecnologias digitais, que têm o potencial de expandir o alcance das iniciativas de saúde pública e melhorar processos. A adoção dessas ferramentas, em combinação com uma governança mais colaborativa e o fortalecimento da formação interprofissional, permitirá que a enfermagem mantenha sua posição de destaque na transformação dos sistemas de saúde. Priorizar a valorização e o aprimoramento dos enfermeiros não só aumenta a eficácia das FESP, mas também ajuda a criar sistemas de saúde mais inclusivos, resilientes e aptos a enfrentar os desafios do século XXI.

Por se tratar de estudo teórico-reflexivo, os achados estão baseados na interpretação crítica dos documentos da OPAS, o que pode limitar a generalização dos resultados. A ausência de dados empíricos também restringe a compreensão das práticas concretas da enfermagem nos diferentes contextos das Américas.

## Data Availability

Os dados da pesquisa estão disponíveis no corpo do artigo.
